# Elevated Liver Enzymes in Patients with Type 2 Diabetes Mellitus and Non-alcoholic Fatty Liver Disease

**DOI:** 10.7759/cureus.3626

**Published:** 2018-11-23

**Authors:** Amrendra Mandal, Bikash Bhattarai, Paritosh Kafle, Mazin Khalid, Saikiran K Jonnadula, Jenny Lamicchane, Rajan Kanth, Vijay Gayam

**Affiliations:** 1 Internal Medicine, Interfaith Medical Center, Brooklyn, USA; 2 Internal Medicine, St. John Riverside Hospital, Yonkers, USA; 3 Gastroenterology, Carilion Clinic, Roanoke, USA

**Keywords:** non-alcoholic fatty liver disease (nafld), type 2 diabetes mellitus (t2dm), alanine aminotransferase (alt), alanine transaminase

## Abstract

Background

Non-alcoholic fatty liver disease (NAFLD) is emerging as the most common chronic liver condition. Approximately 70% of type 2 diabetes mellitus (T2DM) patients have a fatty liver; the progression to non-alcoholic steatohepatitis (NASH) dramatically increases the risks of cirrhosis and hepatocellular carcinoma. The aim of our study was to assess the profile of liver enzymes in subjects with T2DM and NAFLD.

Method

This was a cross-sectional clinic-based study in patients with T2DM. An ultrasonography of the abdomen was done in all patients in order to examine the presence of a fatty liver. Body mass index (BMI), lipid profile, and liver enzymes were also analyzed in all patients. Institutional Review Board (IRB) approval was provided by the National Academy of Medical Sciences, Bir Hospital, Nepal. Unpaired *t*-test, Chi-square/Fisher's exact test (for categorical variables), and the Pearson/Spearman correlation test were used to find a significant difference, association, and correlation between two or more groups, respectively. The Statistical Package for Social Sciences (SPSS)® Statistics, version 16 (IBM SPSS Statistics, Armonk, NY) was used to analyse the data.

Results

The study was carried out in 210 patients, and out of the 210 patients, 119 (56.6%) were male and 91 (43.3%) were female. The patients were divided into two groups, i.e., the normal alanine aminotransferase (ALT) group and the elevated ALT group. The mean age of the patients was 56.28 ± 12.3 years in the normal alanine aminotransferase (ALT) group and 58.6 ± 24.7 in the elevated ALT group. The number of T2DM patients with a fatty liver was 117 (55.7%) and those with a non-fatty liver was 93 (44.2%) based on an ultrasonography scan. Subjects with NAFLD had a significantly higher ALT (p < 0.001) but no significant rise in serum aspartate aminotransferase (AST), gamma-glutamyl transferase (GGT), and alkaline phosphatase (ALP) levels. The area under the receiver operating characteristic (AUROC) curve for the prediction of fatty liver based solely on the ALT was 0.84 with the confidence interval (CI) between 0.76 and 0.92 (p < 0.05).

Conclusions

Non-alcoholic fatty liver disease is highly prevalent in patients with T2DM. Timely diagnosis and management of the abnormal liver parameters may help to minimize liver-related morbidity and mortality in the diabetic population.

## Introduction

Nonalcoholic fatty liver disease (NAFLD) is emerging as the most common cause of chronic liver disease in Western countries, as well as worldwide [1]. It is characterized by hepatic steatosis in the absence of excessive alcohol consumption. The spectrum of NAFLD ranges from simple steatosis to non-alcoholic steatohepatitis (NASH), fibrosis, and cirrhosis with its complications, such as decompensation and hepatocellular carcinoma (HCC) [2]. It is expected to become the most important indication for liver transplantation in the near future.

Most patients with NAFLD are asymptomatic and typically are identified when abnormal liver studies are noted on routine laboratory assessment. In particular, the liver enzymes, alanine aminotransferase (ALT), and aspartate aminotransferase (AST) levels are elevated. However, these enzymes may not be elevated in all cases of NAFLD, and the level of aminotransferases may not reliably predict the extent of inflammation and cirrhosis [3].

Herein, we describe a study performed to investigate the association between liver enzymes and NAFLD in patients with type 2 diabetes mellitus (T2DM) determined by ultrasonography in a Nepalese population.

## Materials and methods

This was a cross-sectional, observational study conducted between September 2009 and December 2010. Patients with a diagnosis of T2DM who were being treated as outpatients at Bir Hospital were enrolled in the study. The study protocol was approved by the institutional review board (IRB) of the National Academy of Medical Science (NAMS), Bir Hospital. All patients who satisfied the inclusion criteria were recruited in the study. The enrolled patients further underwent a structured health history and physical examination, including measurements of body mass index (BMI). All patients had liver function biomarkers, including alanine aminotransferase (ALT), aspartate aminotransferase (AST), alkaline phosphatase (ALP), gamma-glutamyl transferase (GGT), glycosylated hemoglobin (HbA1c), hepatitis B surface antigen (HBsAg), and anti-hepatitis C antibody studies. The normal ranges for the labs were as follows: ALT: 10 - 40 international units per liter (IU/L), AST: 10 - 35 IU/L, GGT: 5 - 30 IU/L, and ALP: 80 - 290 IU/L. The study subjects with or without NAFLD were divided into groups according to the ALT level.

Patients with alcohol consumption, known liver disease, viral hepatitis, and steatogenic medication were excluded. A detailed history of alcohol consumption was taken with critical level being < 20 g/day in women and < 30 g/day in men [4]. Abdominal ultrasound was used as an imaging tool for diagnosing NAFLD [5].

A P value < 0.05 was set as the level of statistical significance. All analysis was performed by using the Statistical Package for Social Sciences (SPSS)® Statistics, version 16 (IBM SPSS Statistics, Armonk, NY).

## Results

**Figure 1:**
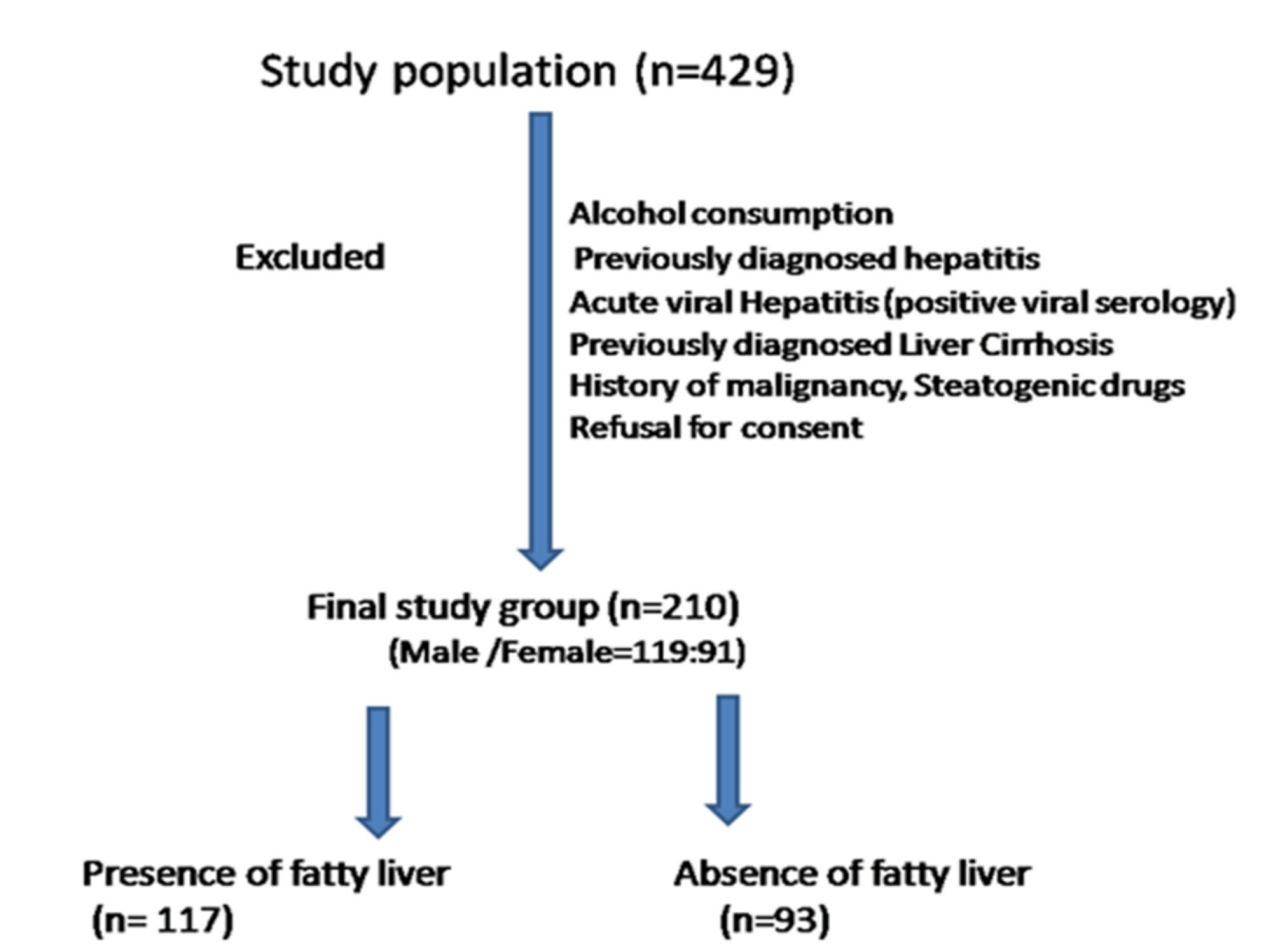
Flow Chart of the Study Populations

The flow diagram of the study populations with T2DM are shown in Figure 1.

The baseline characteristics and liver enzymes of patients with T2DM are shown in Table 1.

**Table 1:** Baseline Characteristics and Liver Enzymes of Patients with Type 2 Diabetes Mellitus (T2DM) BMI: body mass index; Hb A1c: glycosylated hemoglobin; HDL: high-density lipoprotein; n: number; NAFLD: non-alcoholic fatty liver disease; SD: standard deviation

Characteristics	ALT < 40 IU/L	ALT ≥ 40 IU/L	P-value
Age, years (mean ± SD)	56.28 ± 12.3	58.6 ± 24.7	NS
Sex			
Males n (%)	55 (26.1)	64 (30.4)	NS
Females n (%)	42 (20)	49 (23.3)	NS
BMI - Kg/m2, (mean)	28.1 ± 6.7	30.2 ± 7.5	NS
Total cholesterol - mg/dL, (mean)	208	215	NS
HDL - mg/dL (mean)	42	39	NS
Triglyceride - mg/dL (mean)	187	210	NS
Hb A1C (%)	6.9 ± 2.3	7.1 ± 2.5	NS
NAFLD n (%)	32 (27.3)	85 (72.6)	< 0.001

This study was carried out in 210 patients, and of those patients, 119 (56.6%) were male and 91 (43.3%) were female. The patients were divided into two groups, i.e., the normal ALT group and the elevated ALT group. The mean age of the patients was 56.28 ± 12.3 years in the normal ALT group and 58.6 ± 24.7 in the elevated ALT group. The number of T2DM patients with fatty liver was 117 (55.7%) and those with a non-fatty liver was 93 (44.2%) based on ultrasonography scans. Subjects with NAFLD had significantly higher ALT levels (p < 0.001) but no significant rise in AST or GGT levels. The relationship of liver biochemistry and enzymes in T2DM with and without fatty liver based on ultrasonography are shown in Table 2.

**Table 2:** Relationship of Liver Biochemistry and Enzymes in T2DM With and Without Fatty Liver Based on Ultrasonography ALP: alkaline phosphatase; ALT: alanine aminotransferase; AST: aspartate aminotransferase; DM: diabetes mellitus; GGT: gamma-glutamyl transferase; T2DM: type 2 diabetes mellitus

Parameters	DM without fatty liver	DM with fatty liver	p-value
ALT	24.7 ± 7	51.5 ± 27	< 0.001
AST	22 ± 15	26.1 ± 13	0.24
GGT	15 ± 37	23 ± 52	0.09
ALP	132 ± 56	141 ± 60	0.08

**Figure 2:**
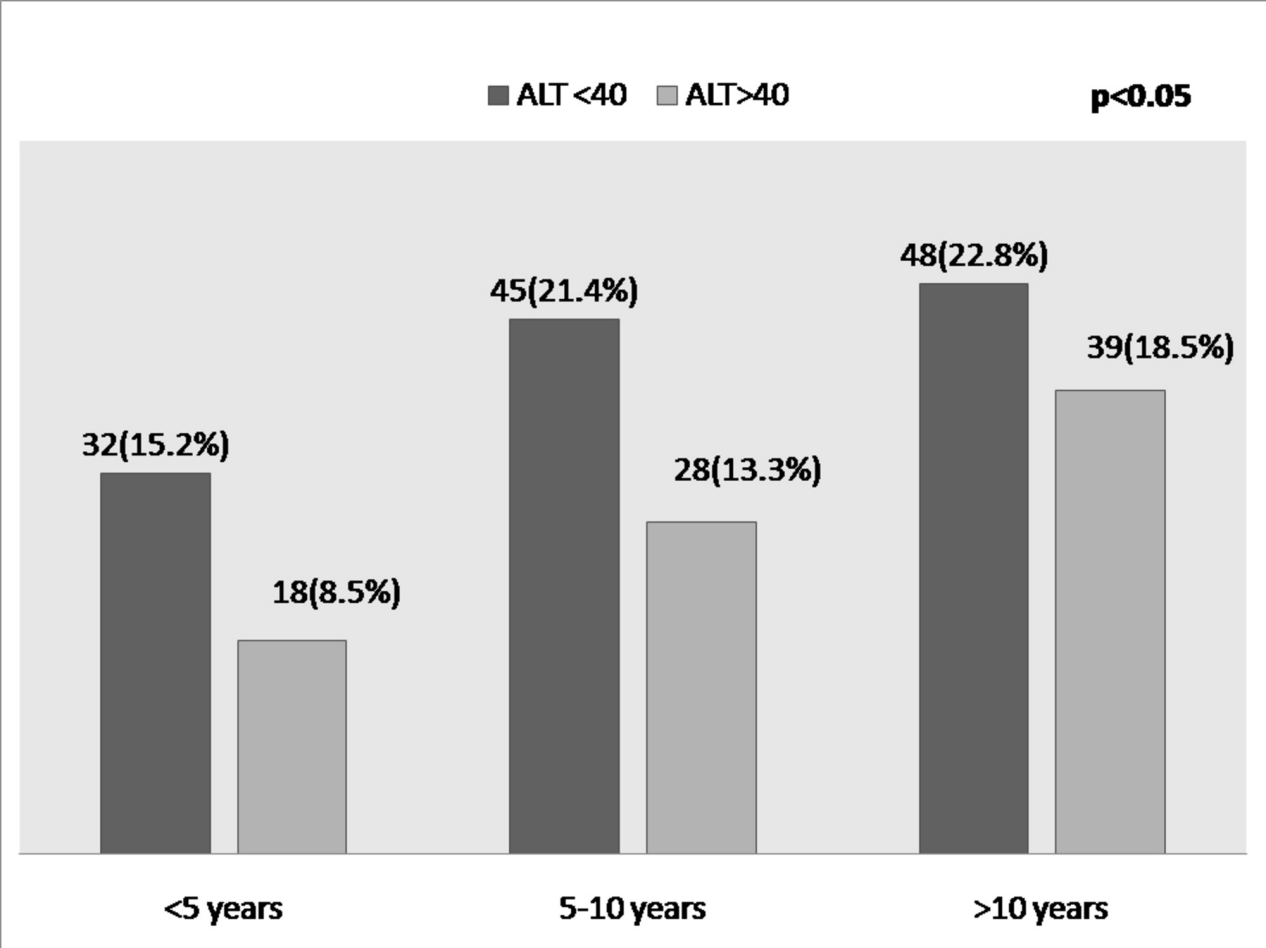
Bar Diagram Showing Correlation of ALT with Duration of T2DM Irrespective of the Presence of NAFLD ALT: alanine aminotransferase; NAFLD: non-alcoholic fatty liver disease; T2DM: type 2 diabetes mellitus

BMI and triglyceride values were higher in fatty liver but were not statistically significant. The duration of T2DM was also significantly associated with elevated liver enzymes, irrespective of the presence of a fatty liver, as shown in Figure 2.

**Figure 3:**
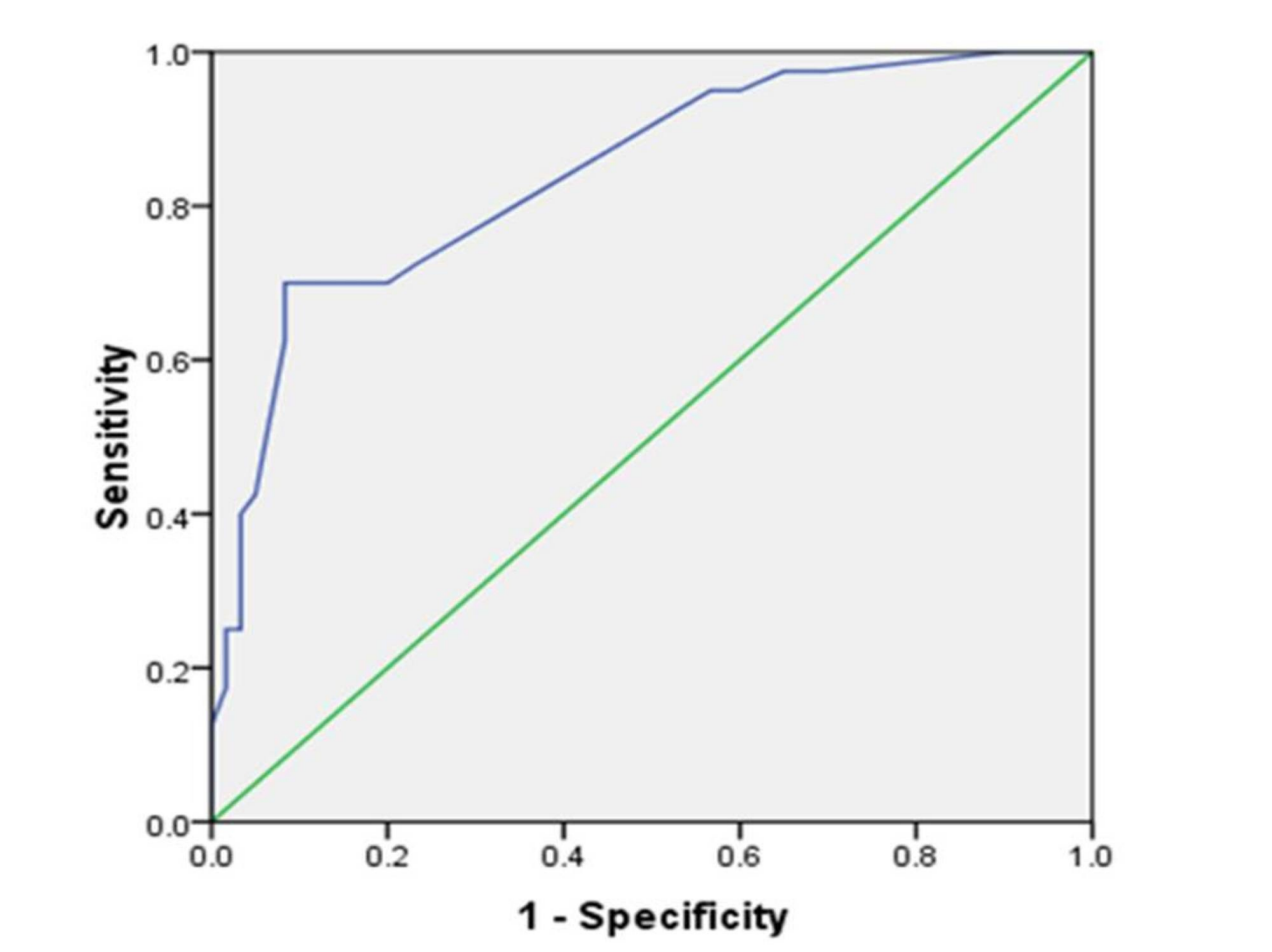
Receiver Operating Character (ROC) Curve for ALT in Predicting the Presence of a Fatty Liver ALT: alanine aminotransferase

The area under the receiver operating characteristic (AUROC) curve for the prediction of fatty liver based solely on the ALT value was 0.84 with a confidence interval (CI) between 0.76 and 0.92 (p < 0.05) (Figure 3).

## Discussion

Our results showed that liver enzymes, especially ALT, were independently associated with NAFLD in a Nepalese population with T2DM.

A higher incidence of liver function test abnormalities has been associated with individuals with T2DM than individuals without T2DM [6]. In the present study, the ALT was elevated in 40.4% of the diabetic population, while the AST and ALP were increased only in 17% and 16% of the diabetic population, respectively. Similar to our study, Shrestha et al. and Thanpari et al. from Nepal also observed a statistically significant increase in ALT levels in diabetic patients as compared to the control groups [7-8]. Likewise, a study conducted by Ni et al. from Malaysia also had a similar pattern with 18%, 12%, and 5% for ALT, AST, and ALP, respectively, in the diabetic population [9]. In the Ghimire et al. study, the values of both transaminases were higher in diabetic females than in male diabetic patients, though not at a statistically significant level [10]. This finding was not observed in our study.

A large United Kingdom study illustrated that NAFLD detected by ultrasonography (USG) was the most common cause of abnormal liver biochemistry [11]. Diabetic subjects with NAFLD had significantly higher ALT, AST, and GGT levels and a significantly lower AST: ALT ratio in comparison with diabetic subjects without NAFLD, but there was no significant difference in the ALP levels. Hence, the controversy relied upon a different increased pattern of liver function tests (LFTs) in several research studies. In addition, a few studies showed AST and some other studies reported GGT as being the most abnormal parameter in their diabetic populations [7-8, 12].

Additionally, there are a number of studies that support significantly elevated levels of transaminases in the diabetic population, especially more so with NAFLD than non-NAFLD, including the present study. The ultrasonography results of 210 patients with T2DM in our study showed fatty infiltration in 55.7% of the patients, which was slightly lower compared to the Prashanta et al., Hernaez et al., and Leite et al. studies with T2DM [13-15]. Higher triglyceride levels were also associated with NAFLD in a study by Leite et al., and among NAFLD patients, they discovered 78% of the patients to have NASH based on the histopathological study [13]. The presence of high triglycerides, low HDL cholesterol levels, and increased ALT levels were independently associated with a higher risk of histologically confirmed non-alcoholic steatohepatitis (NASH). In another study by Leite et al. in 2011, the presence of NASH was independently associated with high serum GGT levels, older age, and male gender [16]. However, Prashanta et al. confirmed NAFLD by liver biopsy in only 54.11% of T2DM patients, which was slightly lower than the Leite et al. study [13, 17]. While the simple fatty liver seen in NAFLD does not correlate with increased morbidity or mortality, the progression to NASH dramatically increases the risks of cirrhosis and hepatocellular carcinoma.

ALT is located in the hepatocellular cytosol, whereas AST is located mostly within the mitochondria. In fact, NAFLD and NASH have been reported to be the most common chronic causes of elevated liver enzymes and are often a clue for the need for further diagnostic evaluation [18]. The mechanism of the development of a fatty liver is explained by the insulin resistance that activates lipolysis, resulting in the accumulation of non-esterified fatty acids. This enhanced fat accumulation in the liver is known to be directly toxic to hepatocytes [19]. This results in an increase in ALT [6]. Patients with NAFLD have increased liver-related and overall mortality commonly due to cardiovascular disease [4]. 

A liver biopsy remains the gold standard for characterizing liver histology in NAFLD, but it is expensive and carries some morbidity and, very rarely, mortality risk. Thus, it should be performed in those who would benefit the most from the diagnostic and prognostic perspectives. Prati et al. have proposed an ALT cut-off of for healthy males and females to be 30 U/L and 19 U/L, respectively, to be diagnostically useful in NAFLD [20]. Considering the lower cut-off based on the Prati et al. study, the sensitivity for ALT would considerably increase in diagnosing NAFLD.

Based on the ROC curve, our study had an AUROC of 0.84 for ALT in predicting NAFLD in diabetic patients. Routine ultrasound examination of the abdomen (for the presence of NAFLD) may be performed in T2DM patients who have elevated liver enzymes, and subsequently, early treatment and reduction of cirrhosis and its complications may be possible.

## Conclusions

Non-alcoholic fatty liver disease is highly prevalent in patients with T2DM. A timely diagnosis and management of the abnormal liver parameters may help to minimize liver-related morbidity and mortality in the diabetic population.
